# Switchgrass *SBP*-*box* transcription factors *PvSPL1* and *2* function redundantly to initiate side tillers and affect biomass yield of energy crop

**DOI:** 10.1186/s13068-016-0516-z

**Published:** 2016-05-05

**Authors:** Zhenying Wu, Yingping Cao, Ruijuan Yang, Tianxiong Qi, Yuqing Hang, Hao Lin, Gongke Zhou, Zeng-Yu Wang, Chunxiang Fu

**Affiliations:** Key Laboratory of Biofuels, Shandong Provincial Key Laboratory of Energy Genetics, Qingdao Institute of Bioenergy and Bioprocess Technology, Chinese Academy of Sciences, Qingdao, 266101 China; Key Laboratory of Biofuels, Qingdao Engineering Research Center of Biomass Resources and Environment, Qingdao Institute of Bioenergy and Bioprocess Technology, Chinese Academy of Sciences, Qingdao, 266101 Shandong China; Biotechnology Research Institute, Chinese Academy of Agricultural Sciences, Beijing, 100081 China; Forage Improvement Division, The Samuel Roberts Noble Foundation, Ardmore, OK 73401 USA

**Keywords:** Energy crop, Biomass, Lignin, SPL transcription factor, Transgenic switchgrass, Tiller initiation

## Abstract

**Background:**

Switchgrass (*Panicum virgatum* L.) is a dedicated lignocellulosic feedstock for bioenergy production. The SQUAMOSA PROMOTER-BINDING PROTEIN (SBP-box)-LIKE transcription factors (SPLs) change plant architecture and vegetative-to-reproductive phase transition significantly, and as such, they are promising candidates for genetic improvement of switchgrass biomass yield. However, the genome-wide identification and functional characterization of *SPL* genes have yet to be investigated in herbaceous energy crops.

**Results:**

We identified 35 full-length *SPL* genes in the switchgrass genome. The phylogenetic relationship and expression pattern of *PvSPLs* provided baseline information for their function characterization. Based on the global overview of *PvSPLs*, we explored the biological function of miR156-targeted *PvSPL1* and *PvSPL*2, which are closely related members of *SPL* family in switchgrass. Our results showed that *PvSPL1* and *PvSPL*2 acted redundantly to modulate side tiller initiation, whereas they did not affect phase transition and internode initiation. Consistently, overexpression of the miR156-resistant *rPvSPL2* in the miR156-overexpressing transgenic plants greatly reduced tiller initiation, but did not rescue the delayed flowering and increased internode numbers. Furthermore, suppression of PvSPL2 activity in switchgrass increased biomass yield and reduced lignin accumulation, which thereby elevated the total amount of solubilized sugars.

**Conclusions:**

Our results indicate that different miR156-targeted *PvSPL* subfamily genes function predominantly in certain biological processes in switchgrass. We suggest that *PvSPL2* and its paralogs can be utilized as the valuable targets in molecular breeding of energy crops for developing novel germplasms with high biofuel production.

**Electronic supplementary material:**

The online version of this article (doi:10.1186/s13068-016-0516-z) contains supplementary material, which is available to authorized users.

## Background

Bioenergy is a renewable energy widely used for heat, electricity, and vehicle fuel in the world [1]. First-generation biofuel mainly derived from starch- or sugar-based ethanol has been commercialized successfully in many countries. In Brazil, for instance, around 25 % of road transport fuel is contributed by bioethanol [2]. However, our current liquid biofuels are produced from food or feed crops which raise concerns about food and feedstock price and land use impacts [3]. Switchgrass (*Panicum virgatum* L.) is a dedicated lignocellulosic feedstock used for next-generation biofuel production in the United States [4]. As a tall-growing perennial C4 grass, switchgrass can grow on land unsuitable for food and feed crops as a low-cost harvest, and its abundant lignocellulosic biomass can be burnt as solid or liquid fuels to meet the growing worldwide demands for energy [5]. Although there are various technologies for conversion of lignocellulosic biomass to biofuel in the research and development phase, biomass improvement of switchgrass is still a cost-effective route to the commercialization of next-generation biofuels [6].

*SQUAMOSA PROMOTER-BINDING PROTEIN-LIKE* (*SPL*) genes encode a class of plant-specific transcription factors containing a highly conserved SQUAMOSA PROMOTER-BINDING PROTEIN (SBP) domain with approximately 78 amino acid residues [7]. SPLs affect diverse developmental processes of plants, such as vegetative-to-floral transition, leaf initiation, shoot/panicle branching, tillering, and male fertility [8–13]. Other studies indicate that SPLs participate in copper homeostasis, cytokinin response, and the biosynthesis of anthocyanin, terpene, carotenoid, and lignin [14–19]. These results suggest that SPLs are a group of functionally diverse transcription factors, which is consistent with SPLs representing a structurally heterogeneous family. Genome-wide identification of *SPLs* has been carried out in many plant species such as Arabidopsis, citrus, cotton, grape, poplar, and rice [20–25]. The members of *SPLs* in the above six plant species vary in number from 15 to 28, and the deduced amino acid sequences varies from 130 to 1140 in length. Strikingly, about two-thirds of these *SPLs* in each genome contain the complementary sites of miR156, suggesting a critical role of miR156/SPLs module in the internal controls of plant growth and development [26, 27]. Previous work has shown that the expression levels of miR156 are gradually increased during rice leaf development, and consistently the lower expression levels of five miR156-targeted *OsSPLs* are determined in old leaves compared with the young ones [28]. Overexpression of miR156 in rice downregulates the transcript abundance of the above *SPLs* strongly, disrupts the temporal expression patterns of these *OsSPLs*, and leads to delayed flowering, reduced plant height, and small panicle size [25, 28]. In addition, our previous work has revealed that the expression levels of eight miR156-targeted *SPLs* are differentially influenced in miR156b-overexpressing transgenic switchgrass plants [29]. The reduction in transcript abundance of these *SPLs* accompanying with the alteration of morphological characterization depends on the level of miR156 in the transgenic switchgrass plants. These results suggest that it is required to maintain the expression of different *SPL* genes at appropriate levels in vivo for plant normal development and healthy growth.

Overexpression of miR156 in plants can lead to diverse morphological characterization including increased tiller/shoot branching, elevated leaf initiation rate, and delayed flowering [8, 25, 28–31]. These results implicate that the miR156/SPLs module is a potential target for biomass improvement of energy crops. The strategy currently employed for constitutive overexpression of miR156, however, cannot exactly distinguish the contribution of each *SPL* to a combination of phenotypes. It has been shown that more than one thousand probe sets have altered expression in the rice or switchgrass miR156-overexpressing transgenic plants, which is consistent with the nature of *SPLs* as the functionally diverse transcription factors [28, 29]. Moreover, previous studies have demonstrated that overexpression of miR156 in rice leads to reduced plant height and small panicle size, which are negative traits for grain production [25]. Other studies indicate that the miR156b-overexpressing transgenic switchgrass lines can be classified into three groups based on their molecular and morphological characterization and only the group I and group II plants have significantly increased biomass and fermentable sugar yield. On the contrary, the group III plants with the highest miR156 accumulation show dwarfism and low biomass in spite of a dramatic increase in tiller number [29]. These studies suggest that the plants with high miR156 levels can cause severe morphological changes that impair the positive effects of the increased tiller/shoot number on total biomass. Thus, it is worth clarifying the detailed function of the individual *SPL*, which will avoid the mutual interference among different *SPL* subfamily genes. To date, the molecular identification and functional characterization of SPL family, however, have yet to be investigated in any herbaceous bioenergy crops.

Genome-wide identification revealed 35 full-length *SPLs* in the switchgrass genome, 21 of which contain the complementary sequences of miR156. The expression levels of the miR156-targeted *PvSPLs* were gradually decreased during leaf and internode development. A reverse genetic analysis of *PvSPL2* indicates that *PvSPL2* can control side tiller initiation and internode elongation predominantly, whereas they had no effects on flowering time and internode initiation in switchgrass. Finally, genetic manipulation of PvSPL2 in switchgrass increased tiller numbers and impaired lignin biosynthesis, and therefore significantly improved switchgrass biomass yield and cell wall saccharification efficiency.

## Results

### Genome-wide identification, phylogenetic relationship, and chromosomal distribution of *PvSPLs*

**Fig. 1:**
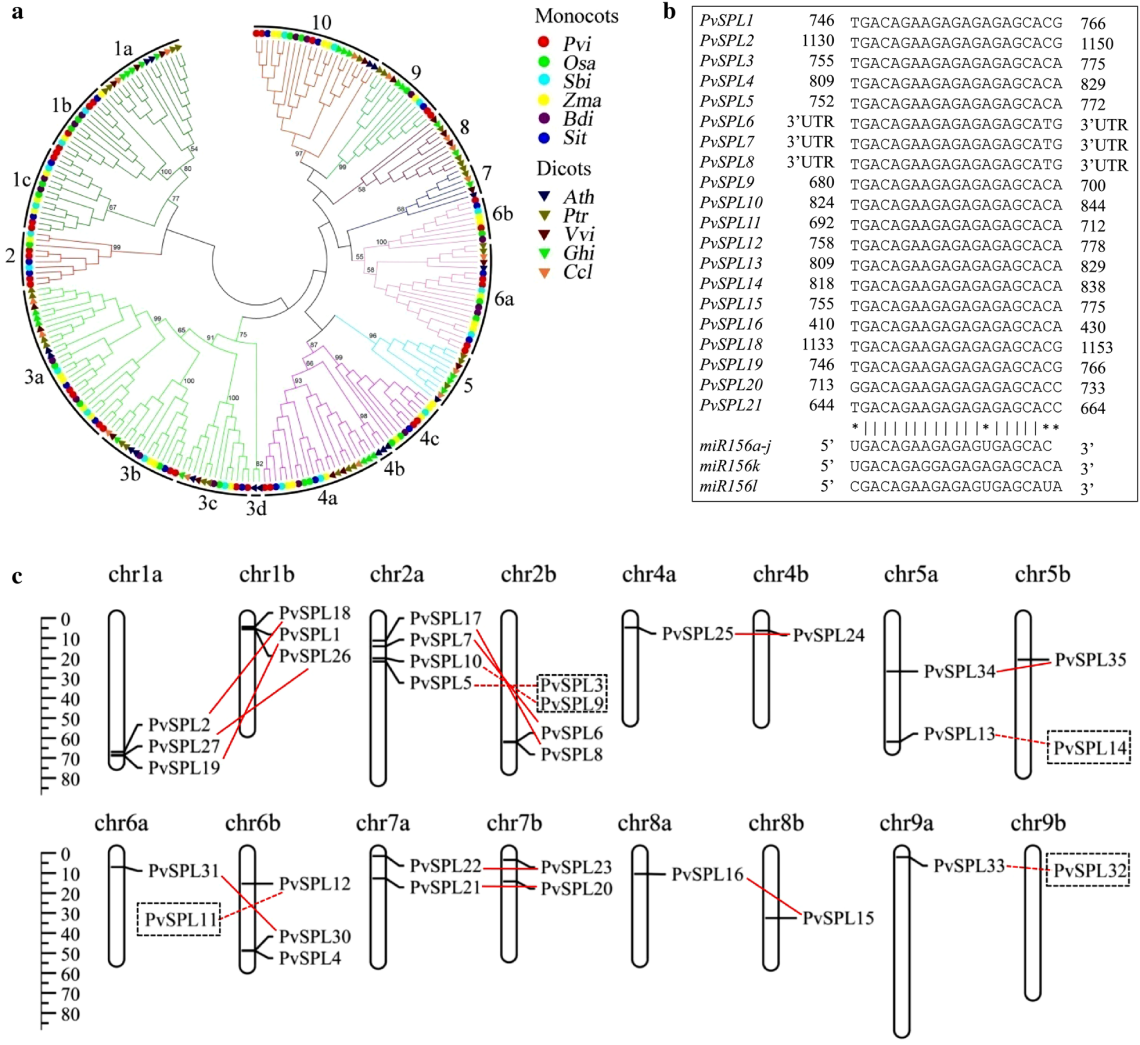
Genome-wide identification of *PvSPLs* in switchgrass. **a** Phylogenetic relationship of SPLs. A maximum likelihood tree was constructed in PhyML version 3.0 on the basis of multiple alignments of SBP domain sequences from monocot species (switchgrass, foxtail millet, maize, sorghum, rice, and Brachypodium) and dicot species (Arabidopsis, citrus, cotton, grape, and poplar). *Pvi* switchgrass, *Sit* foxtail millet, *Zma* maize, *Sbi* sorghum, *Osa* rice, *Bdi* Brachypodium, *Ath* Arabidopsis, *Ccl* citrus, *Ghi* cotton, *Vvi* gape, and *Ptr* poplar. **b** Sequence alignment of miR156 mature sequences with complementary sequences of *PvSPLs*. The *dots* between miR156 and targeted *PvSPL* sequences indicate mismatches. **c** Chromosomal distribution of *PvSPLs* in the switchgrass genome

Based on the Hidden Markov Model profile of conserved SBP domain (PF03110) sequence, 35 *SPL* family members, designated as *PvSPL1* to *PvSPL35*, were identified in the switchgrass genome using the Blastp and tBlastn programs. The deduced protein sequences of these *PvSPLs* ranged from 180 (PvSPL6) to 1113 (PvSPL31) amino acids in length, and the isoelectric point varied from 5.28 (PvSPL29) to 10.27 (PvSPL7) (Additional file 1: Table S1). The SPL sequences identified from six genome-sequenced grasses (switchgrass, foxtail millet, maize, sorghum, rice, and Brachypodium) and five dicot species previously studied (Arabidopsis, citrus, cotton, grape, and poplar) were downloaded for the phylogenetic relationship analysis. A maximum likelihood (ML) tree was constructed in PhyML version 3.0 on the basis of multiple alignments of full-length SPL sequences (Fig. 1a). The phylogenetic tree revealed that the SPLs can be classified into 10 orthologous groups (OGs) according to the tree topologies and bootstrap values (>50 %). The switchgrass PvSPLs were clustered into seven OGs (Fig. 1a; Additional file 2: Table S2). Twenty-one out of thirty-five *PvSPLs* contained the nearly complementary sequences of miR156 in the coding region or 3′-untranslated region (Fig. 1b). These miR156-targeted PvSPLs including eight previously studied belonged to OG1, 2, 4, 9, and 10 (Additional file 2: Table S2). The PvSPLs from the same clade had the similar exon/intron arrangement and motif distributions (Additional file 3: Figure S1). Strikingly, the monocot and dicot SPLs distinctly diverged into different OGs or clusters within OGs (Fig. 1a). For example, all SPL members of OG5, 7, and 8 were from dicot species, while all members of OG2 belonged to monocot species including PvSPL6/7 and 8/17. Similarly, the monocot SPL members clustered together and clearly diverged from their corresponding dicot orthologs in other OGs (Fig. 1a; Additional file 2: Table S2). Taken together, these results indicate that *SPL* genes were duplicated after the divergence between monocots and dicots.

To examine the chromosomal distribution of *PvSPLs*, the physical locations of all *PvSPLs* on chromosomes (Chrs) were obtained through Blastn searches against switchgrass genome database in Phytozome (Fig. 1c). The lowland switchgrass cultivars are allotetraploid (2n = 4x = 36) and consist of two highly homologous subgenomes, designated as Chr a and Chr b. The *PvSPLs* existed as paralogous gene pairs in the switchgrass genome with only one exception (*PvSPL4*), and 33 *PvSPLs* were mapped on all chromosomes except Chr 3. According to the chromosome locations, no tandem repeat *SPL* genes were found in switchgrass.

### Expression patterns of *PvSPLs* in wild-type switchgrass plants

**Fig. 2:**
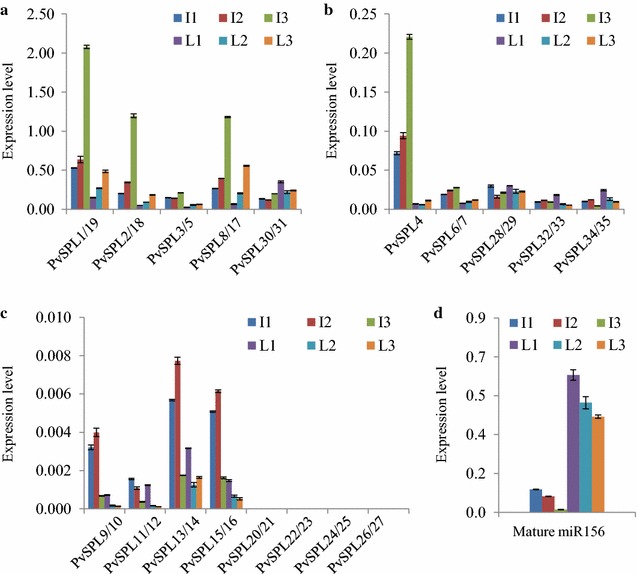
Expression patterns of *PvSPLs* in wild-type switchgrass plants. **a**–**c** Expression patterns of *PvSPLs* in wild-type switchgrass plants were revealed by qRT-PCR. Switchgrass *Ubq2* was used as the reference for normalization. **d** Expression patterns of mature miR156 in wild-type switchgrass plants were detected and quantified by a highly sensitive quantitative real-time PCR method. miRNA168 was used as the reference for normalization. Values are mean ± SE (*n* = 3)

Switchgrass tiller consists of nodes, internodes, leaf blades, leaf sheaths, and inflorescence, of which internodes and leaves contribute to the majority of the above-ground biomass after harvest. To study whether *PvSPLs* are involved in the development of internode and leaf, the expression levels of the switchgrass *SPL* gene family members were examined by the quantitative reverse transcription polymerase chain reaction (qRT-PCR). Our results showed that 9 of the 17 *PvSPL* gene pairs (*PvSPL1/19*, *2/18*, *3/5*, *6/7*, *8/17*, *28/29*, *30/31*, *32/33*, and *34/35*) and *PvSPL4* had relatively high expression levels in internode and leaf compared with the other *PvSPLs* (Fig. 2a–c). Among the low-expressing *PvSPLs*, the expression levels of four *PvSPL* gene pairs (*PvSPL20/21*, *22/23*, *24/25*, and *26/27*) were under detection limit (Fig. 2c). Moreover, we studied the expression patterns of *PvSPLs* in the successive internodes and leaves along the tiller. Our results revealed that those highly expressing miR156-targeted *PvSPLs* displayed gradually decreased expression during the development of internode and leaf, whereas other non-miR156-targeted *PvSPLs* did not show the similar pattern except *PvSPL32/33* and *PvSPL34/35* which displayed an opposite pattern in leaf development (Fig. 2a, b). Given the fact that miR156 directly regulates the expression levels of its *SPL* targets, we next decided to examine miR156 expression pattern in internodes and leaves. Our results showed that the levels of mature miR156 in switchgrass were gradually increased during the development of internode and leaf (Fig. 2d). In addition, internodes contained lower mature miR156 levels than leaves, which was consistent with the finding that the relatively high transcript abundances of the miR156-targeted *PvSPLs* were observed in internodes compared with leaves (Fig. 2a–c).

### Expression patterns of *PvSPLs* in miR156b-overexpressing transgenic switchgrass plants

**Fig. 3:**
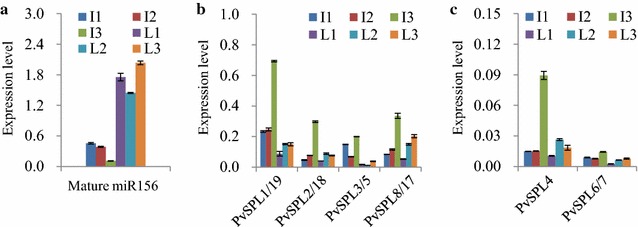
Expression patterns of *PvSPLs* in miR156-overexpressing transgenic switchgrass plants. **a** Expression patterns of mature miR156 in transgenic switchgrass plants were detected and quantified by a highly sensitive quantitative real-time PCR method. miRNA168 was used as the reference for normalization. **b**–**c** Expression patterns of *PvSPLs* were revealed by qRT-PCR. Switchgrass *Ubq2* was used as the reference for normalization. Values are mean ± SE (*n* = 3)

We have previously studied eight miR156-targeted *PvSPLs* (*PvSPL1*-*8*) exhibiting reduced expression in miR156-overexpressing transgenic switchgrass plants. To study whether the temporal expression patterns of the miR156-targeted *PvSPLs* observed in wild type were altered by miR156 overexpression, we first examined the levels of miR156 in an miR156 highly overexpressing transgenic line TmiR156OE9. Our results revealed that the temporal expression pattern of mature miR156 was disappeared in the leaves of transgenic switchgrass plants, whereas it was still well maintained in internodes (Fig. 3a). Furthermore, we determined the expression levels of the miR156-targeted *PvSPLs* in the miR156 overexpressor. Our results showed that overexpression of miR156 substantially reduced the transcript abundances of its *SPL* targets in both internodes and leaves, whereas it did not alter the expression patterns during the internode development (Fig. 3b, c). Consistently, the disrupted miR156 expression pattern in leaves altered the temporal expression patterns of the miR156-targeted *PvSPLs* except *PvSPL6/7* and *PvSPL8/17*, suggesting that other unknown mechanisms may participate in regulating expression levels of certain *PvSPLs* (Fig. 3).

### Identification of the potential *PvSPLs* for switchgrass biomass improvement

Tiller density is an important trait for breeding high-yielding cultivars of herbaceous energy crops. The representative miR156-targeted *PvSPL2*, *3,* and 6 from OG4, 10, and 2 exhibited high expression levels during tiller development and were therefore focused to study their potential roles in controlling tiller density (Figs. 1, 2). We first examined the relationship between the expression levels of these miR156-targeted *PvSPLs* and tiller numbers using 12 independent positive miR156 overexpressors. We found that the transcript abundances of *PvSPL2* had a strong negative correlation (*R*^2^ = 0.82) with till numbers (Additional file 4: Figure S2). Furthermore, we retrieved the closely related paralogs of PvSPL2/18 and PvSPL1/19 from OG4 and found that the expression levels of PvSPL1/19 also negatively correlated (*R*^2^ = 0.70) with till numbers of the miR156-overexpressing transgenic switchgrass plants, suggesting that PvSPL1 and 2 may function to initiate side tillers in switchgrass (Additional file 4: Figure S2). Thus, we chose *PvSPL2* and its closely related paralog *PvSPL1* for further functional characterization.

### Overexpression of a PvSPL2 chimeric repressor in switchgrass

The chimeric repressor gene silencing technology (CRES-T) is a powerful tool for investigation of functionally redundant transcription factors [32]. An EAR repression domain (SRDX) was fused to PvSPL2, and the chimeric PvSPL2SRDX repressor vector was constructed. Ten independent positive PvSPL2SRDX overexpressors generated by *Agrobacterium*-mediated transformation were confirmed by genomic PCR for the insertion of PvSPL2SRDX. Three control plants were produced with pANIC6B empty vector from the same batch of experiment.

**Fig. 4:**
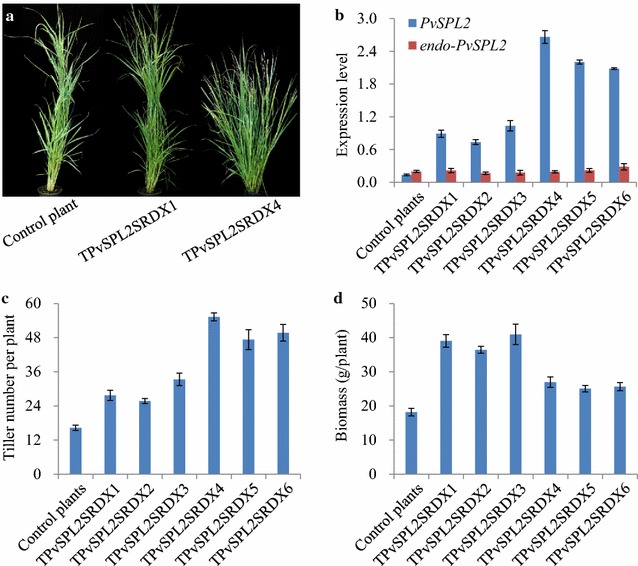
Overexpression of a PvSPL2 chimeric repressor in switchgrass. **a** Morphological characterization of PvSPL2SRDX-overexpressing transgenic switchgrass plants. Representative plants from group I (TPvSPL2SRDX1) and group II (TPvSPL2SRDX4) are shown. Control plant was produced with pANIC6B empty vector from the same batch of experiment. **b** Expression levels of *PvSPL2* in transgenic switchgrass plants were revealed by qRT-PCR. Switchgrass *Ubq2* was used as the reference for normalization. *PvSPL2*: sum of *exo*- and *endo*-*PvSPL2* transcript versions. **c** Tiller number of transgenic switchgrass plants. **d** Dry matter biomass yield of transgenic switchgrass plants. The transgenic and control plants were harvested after 4-month growth in the greenhouse. Values are mean ± SE (*n* = 3)

Based on the characterization of tiller development, the transgenic plants were classified into two groups. The morphology of representative plants from each group is illustrated in Fig. 4a. All transgenic lines showed increased tiller numbers under the greenhouse conditions. Six of the ten transgenic lines showed normal tiller height and moderately increased tiller numbers compared with control plants and were classified into group I. Four transgenic lines with highly increased tiller numbers exhibited semi-dwarfism and therefore were assigned to group II. Moreover, analysis of *PvSPL2* expression levels (sum of *exo*- and *endo*-*PvSPL2* transcript versions) by qRT-PCR revealed 5.4- to 19.6-fold overexpression in the transgenic lines, which was consistent with their morphological characterization (Fig. 4b). In contrast, the *endo*-*PvSPL2* expression levels of transgenic lines were comparable with those of control plants (Fig. 4b).

### Morphological characterization of PvSPL2SRDX-overexpressing transgenic switchgrass plants

A total of six transgenic lines representing morphological variations among the transgenic plants from groups I and II were chosen for further detailed analyses. Of them, lines TPvSPL2SRDX-1, -2, and -3 were from group I; TPvSPL2SRDX-4, -5, and -6 from group II. Furthermore, the tiller number, tiller height, internode number, leaf blade length and width, and flowering time of the transgenic lines were measured. The group I transgenic lines showed normal growth and development, but had 1.6- to 2.0-fold increase in tiller numbers compared with control plants (Fig. 4a, c). The group II transgenic lines exhibited 2.9- to 3.4-fold increase in tiller numbers as well as semi-dwarfism and significantly reduced length in internodes and leaf blades (Fig. 4a, c; Table 1). The semi-dwarf transgenic switchgrass plants, however, resembled control plants in both internode number and flowering time (Table 1). Furthermore, we measured dry matter biomass of the PvSPL2SRDX overexpressors. The group I transgenic lines showed 2.0- to 2.3-fold increase in biomass after 4 months of growth. Strikingly, the semi-dwarf lines in group II exhibited an approximately 1.4-fold increase in biomass compared with control plants (Fig. 4d).

**Table 1:** Morphological characterization of PvSPL2SRDX-overexpressing transgenic switchgrass plants

	Plant height (cm)	Internode length (cm)	Internode diameter (mm)	Internode number	Leaf blade length (cm)	Leaf blade width (cm)	Flowering time (day)
Control plants	136.7 ± 5.7	17.9 ± 0.8	2.31 ± 0.19	4–5	30.1 ± 3.3	0.79 ± 0.07	108 ± 4
TPvSPL2SRDX1	130.1 ± 4.4	18.8 ± 0.5	2.07 ± 0.33	4–5	28.3 ± 1.3	0.64 ± 0.10	107 ± 6
TPvSPL2SRDX2	137.9 ± 6.1	19.3 ± 2.0	1.93 ± 0.47	4–5	34.7 ± 3.7	0.74 ± 0.09	99 ± 2
TPvSPL2SRDX3	132.8 ± 7.3	17.1 ± 1.3	2.27 ± 0.09	4–5	37.2 ± 4.0	0.82 ± 0.13	100 ± 5
TPvSPL2SRDX4	83.2 ± 4.7*	13.6 ± 0.7*	2.43 ± 0.27	4–5	20.3 ± 1.7*	0.87 ± 0.17	106 ± 4
TPvSPL2SRDX5	78.7 ± 5.8*	11.7 ± 1.1*	2.70 ± 0.37	4–5	21.2 ± 1.5*	0.81 ± 0.07	103 ± 3
TPvSPL2SRDX6	81.5 ± 6.9*	12.9 ± 1.5*	2.38 ± 0.13	4–5	19.0 ± 2.3*	0.84 ± 0.05	107 ± 2

Plant height of switchgrass was measured after 4-month growth in the greenhouse. The 4-month-old tillers were used to measure internode length (internode 3), internode diameter (internode 3), internode number, and leaf blade length and width. Five tillers were measured for each replicate. Control plants were produced with pANIC6B empty vector from the same batch of experiment. Values are mean ± SE (*n* = 3)

* Significance corresponding to *P* < 0.05 (One-way ANOVA, Dunnett’s test)

### Effect of PvSPL2SRDX overexpression on lignin accumulation

**Fig. 5:**
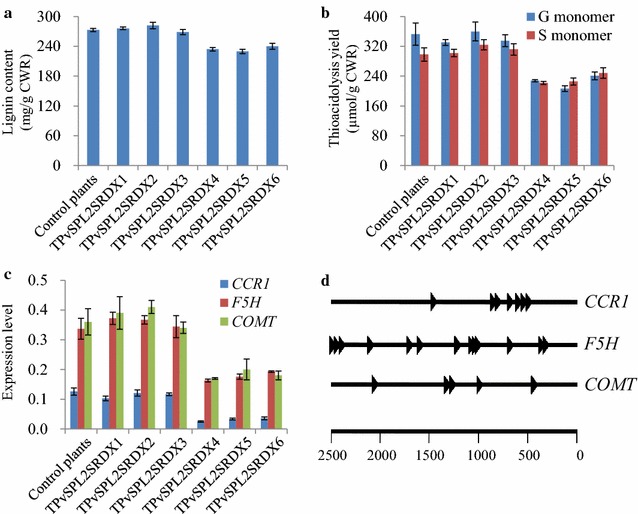
Effects of PvSPL2SRDX overexpression on lignin accumulation. **a** AcBr lignin content of transgenic switchgrass plants. **b** Thioacidolysis yield of transgenic switchgrass plants. The transgenic and control plants were harvested after 4-month growth in the greenhouse. *CWR* cell wall residue. **c** Transcript abundance of monolignol genes in transgenic switchgrass plants. Values are mean ± SE (*n* = 3). **d** SBP core binding sites located in the promoter sequences of the related monolignol genes. ►, GTAC

To study whether repression of PvSPL2 activity affects lignin biosynthesis, we determined lignin content and lignin composition of PvSPL2SRDX overexpressors. No changes in lignin content and composition were found in group I transgenic plants. In contrast, the acetyl bromide (AcBr) lignin content in group II transgenic plants showed up to 12.2–16.0 % reduction compared with control plants (Fig. 5a). Moreover, lignin composition analysis by thioacidolysis method revealed a significant reduction in both guaiacyl (G) and syringyl (S) lignin monomer yield in group II transgenic plants (Fig. 5b). We further investigated the expression levels of monolignol genes, of which *4*-*coumarate CoA ligase1* (*4CL1*), *cinnamoyl CoA reductase1* (*CCR1*), *ferulate 5*-*hydroxylase* (*F5H*), *caffeic acid O*-*methyltransferase* (*COMT*), and *cinnamyl alcohol dehydrogenase* (*CAD*) have been confirmed to function in lignin biosynthesis of switchgrass [33]. We found that *CCR1*, *F5H*, and *COMT* showed significant reduction in their transcript abundances in group II transgenic plants (Fig. 5c). As a core binding site of SPLs, GTAC motif has been identified to exist in the promoter region of all SPL targets [34]. Thus, we downloaded 2.5 kb promoter sequences of *CCR1*, *F5H*, and *COMT* from phytozome and retrieved more than five GTAC motifs from the promoter region of each gene, suggesting that PvSPL2 could affect lignin biosynthesis through directly binding lignin genes to control their expression (Fig. 5d).

### Effects of r*PvSPL2* overexpression on morphological characterization of switchgrass

**Fig. 6:**
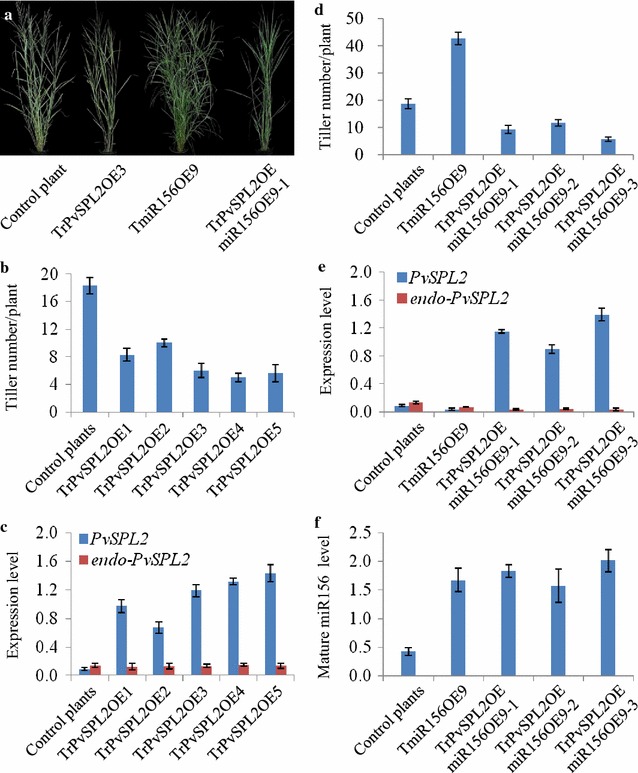
Overexpression of an miR156-resistant rPvSPL2 in switchgrass. **a** Morphological characterization of miR156-, rPvSPL2-, and rPvSPL2 miR156-overexpressing transgenic switchgrass plants. Control plant was produced with pANIC6D empty vector from the same batch of experiment. **b** Tiller number of rPvSPL2-overexpressing transgenic switchgrass lines. **c** Expression levels of *PvSPL2* in rPvSPL2-overexpressing transgenic switchgrass lines were revealed by qRT-PCR. Switchgrass *Ubq2* was used as the reference for normalization. *PvSPL2*: sum of *exo*- and *endo*-*PvSPL2* transcript versions. **d** Tiller number of rPvSPL2 miR156-overexpressing transgenic lines. **e** Expression levels of *PvSPL2* in rPvSPL2 miR156-overexpressing transgenic lines were revealed by qRT-PCR. Switchgrass *Ubq2* was used as the reference for normalization. *PvSPL2*: sum of *exo*- and *endo*-*PvSPL2* transcript versions. **f** Expression levels of mature miR156 in rPvSPL2 miR156-overexpressing transgenic lines were detected and quantified by a highly sensitive quantitative real-time PCR method. miRNA168 was used as the reference for normalization. Values are mean ± SE (*n* = 3)

To further confirm that PvSPL2 functions in till initiation predominantly, we generated five independent positive transgenic switchgrass lines with overexpression of an miR156-resistant *rPvSPL2*. Three control plants were produced with pANIC6D empty vector from the same batch of experiment. All rPvSPL2-overexpressing transgenic lines exhibited normal growth and development, whereas they showed 45–73 % reduction in tiller numbers per plant under the greenhouse conditions (Fig. 6a, b). The transcript abundances of *PvSPL2* (sum of *exo*- and *endo*-*PvSPL2* transcript versions) were significantly increased (7.3- to 15.6-fold of control) in transgenic lines, which was congruent with till numbers. In contrast, the *endo*-*PvSPL2* levels were not changed in transgenic lines (Fig. 6c). To further elucidate that PvSPL2 predominantly affects tiller number in switchgrass, we overexpressed *rPvSPL2* in TmiR156OE9 which was a characteristic miR156-overexpressing transgenic line. Morphological characterization of the transgenic lines showed significant reduction in tiller numbers (13–27 % of TmiR156OE9 and 31–63 % of control) compared to both TmiR156OE9 and control plants, whereas their plant height and flowering time resembled those of TmiR156OE9 transgenic plants, supporting our hypothesis that *PvSPL2* mainly affects tiller numbers in switchgrass (Fig. 6a, d). Three independent positive TrPvSPL2miR156OE9 transgenic lines were subjected to qRT-PCR analysis, revealing a substantial increase (10.4- to 16.0-fold of control) in transcript abundance of *rPvSPL2* in transgenic plants (Fig. 6e). The expression levels of mature miR156 and its targeted *endo*-*PvSPL2*, however, were still severely suppressed (Fig. 6e, f).

### PvSPL1 and PvSPL2 function redundantly to initiate side tillers in switchgrass

PvSPL1 was the closely related paralog of PvSPL2 in switchgrass, which shares 48 % overall amino acid identity and 81 % identity within the SBP-box domain with PvSPL2. Both of them belonged to OG4 subfamily and contained the nearly complementary sequence of miR156 (Fig. 1). To elucidate the function of PvSPL1, we generated PvSL1SRDX-overexpressing transgenic plants (Additional file 5: Figure S3). These plants showed similar morphological characterization to PvSPL2SRDX overexpressors (Additional file 6: Table S3). Moreover, overexpressing an miR156-resistant rPvSPL1 in wild-type and TmiR156bOE9 plants resulted in substantially reduced tiller numbers as well (Additional file 6: Table S3). Taken together, our results suggest that PvSPL1 acts redundantly with PvSPL2 to modulate side tiller initiation in switchgrass.

### Effect of PvSPL2SRDX overexpression on cell wall saccharification

**Fig. 7:**
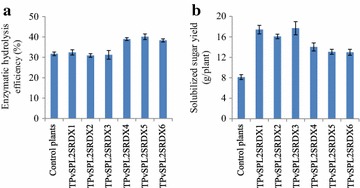
Effects of PvSPL2SRDX overexpression on cell wall conversion. **a** Cell wall enzymatic hydrolysis efficiency of transgenic switchgrass plants. **b** Solubilized sugar yield of transgenic switchgrass plants. The transgenic and control plants were harvested after 4-month growth in the greenhouse. Values are mean ± SE (*n* = 3)

Lignocellulosic biomass is highly recalcitrant to cell wall saccharification due to the presence of lignin. Given the lignin content reduction, we decided to examine cell wall saccharification efficiency of PvSPL2SRDX overexpressors. Sugar release analysis revealed that there was up to a 20.8–26.5 % increase in saccharification efficiency from group II transgenic lines, and no significant difference from group I transgenic lines relative to the control plants (Fig. 7a). The group I transgenic plants, however, still produced 98–117 % more total sugar than the control plants because of high biomass yields (Fig. 7b).

## Discussion

Plant architecture and vegetative-to-floral phase transition are largely determined by the miR156/*SPLs* module, manipulation of which can improve biomass yield of herbaceous bioenergy crops. As the crucial traits in biomass production for biofuels, tiller density and flowering time have attracted much attention from breeders. It had been reported that cultivars with high tiller density and a long duration of vegetative growth are apt to achieve highest biomass yields [35–37]. The miR156-targeted *SPLs*, as plant-specific transcription factors, participate in a broad range of developmental processes in plants. Genome-wide identification of *SPLs* has been studied in numerous dicot and a few monocot species [26, 27]. The gene numbers, molecular characterization, and biological functions of *SPLs* in herbaceous bioenergy crops, however, are largely unknown.

We identified a total of 35 *PvSPLs* in the switchgrass genome, of which 21 *PvSPLs* contained the target sites of miR156. The *PvSPLs* distributed unevenly on nine pairs of chromosomes and existed as paralogous gene pairs except *PvSPL4*. Their variable sizes, complicated gene structure, and various conserve motifs suggest a comprehensive evolution of *SPL* genes during plant responses to various external and internal factors [26, 27]. The switchgrass *SPL* genes were subdivided into seven orthologous groups based on their amino acid sequences. The miR156-targeted *PvSPLs* exhibiting high expression levels during tiller development belonged to OG2, 4, and 10. The members of OG4 and 10 also include the orthologs from Arabidopsis (*AtSPL2/10/11* and *9/15*), rice (*OsSPL14*), and maize (*tsh4*, *ub2*, and *3*), which have been suggested to affect shoot apical dominance and vegetative/reproductive branching [10–12, 38, 39]. The expression pattern analyses indicate that *PvSPL4* and other six miR156-targeted *PvSPL* gene pairs were gradually decreased during the development of internode and leaf in switchgrass. Consistently, mature miR156 exhibited an opposite expression pattern in the above organs. Moreover, the temporal expression patterns of both miR156 and its *PvSPL* targets were strictly controlled in internodes of both wild type and miR156 overexpressors, suggesting that the miR156/*SPLs* module participates in the fine-tuning of stem development.

Overexpression of miR156 in switchgrass can elevate biomass yield and fermentable sugar amount because of greatly increased tiller numbers and severely delayed flowering time [29, 30]. Due to miR156 overexpression, the transcript abundances of miR156-targeted *PvSPLs* were extensively reduced in transgenic switchgrass plants. In spite of the presence of complementary sequences of miR156 in all of *PvSPLs* targets, the reduction rate of each individual *PvSPL* was varied among the transgenic lines, which led to the coordinated effects on switchgrass phenotype [29]. *PvSPL1* and *2* currently identified as the candidates for biomass manipulation belonged to OG4 subfamily, the members of which contain miR156 target site and whose functions remain largely unknown in monocot species. The Arabidopsis orthologs of PvSPL1 and 2, namely AtSPL2, 10, and 11, have been suggested to influence shoot apical dominance redundantly, and suppression of their activity can produce more side shoots [10]. Our previous work has shown that *PvSPL2* can be extensively downregulated in all miR156-overexpressing transgenic switchgrass lines [29]. In this work, a strong negative correlation between *PvSPL1/2* expression levels and tiller numbers was found in the miR156-overexpressing transgenic switchgrass lines, and therefore we postulate that *PvSPL1* and *2* may function side tiller initiation in switchgrass. The hypothesis is supported by our findings that suppression of either PvSPL1 or PvSPL2 activity in switchgrass resulted in a dramatic increase in tiller numbers, whereas overexpression of rPvSPL1/2 had an opposite effect. Additionally, previous studies have shown that overexpression of miR156 can completely inhibit flowering of both greenhouse and field-grown transgenic switchgrass plants [29, 30]. Consistent with these findings, overexpression of miR156-resistant *AtSPL3* can promote early flowering in Arabidopsis [8]. These results suggest that some miR156-targeted *SPLs* have a positive effect on the vegetative-to-floral transition. However, our results showed that overexpressing *rPvSPL1/2* did not shorten the floral transition, indicating that *PvSPL1* and *2* predominantly regulate side tiller initiation rather than flowering in switchgrass. The above conclusion was further supported by the finding that overexpressing *rPvSPL2* in the miR156 overexpressor greatly reduced tiller numbers, but barely rescued the delayed flowering. Moreover, these data suggest that there may be another miR156-targeted *PvSPL* subfamily regulating switchgrass floral transition predominantly. In future studies, it would be interesting to investigate whether the flowering-related *PvSPL*s may affect side tiller initiation as well.

Another typical defect of plant development with highly overexpressed miR156 is the dwarfism due to stunted stem elongation. It has been suggested that miR156 overexpression in switchgrass negatively controls internode elongation, but positively regulates internode numbers [29, 30]. However, it remains to be elucidated if the increased internode numbers account for the reduced internode length in switchgrass miR156 overexpressors. Our results showed that highly repressing PvSPL2 activity impaired internode elongation, but did not affect internode number, clearly suggesting that internode elongation and internode initiation are fine-tuned by distinct factors in switchgrass. Obviously, PvSPL2 and its paralogs are involved in the regulation of stem elongation. Moreover, the limited restoration of rPvSPL2 to the miR156-overexpressing phenotype indicates that both vegetative-to-floral phase transition and internode initiation are mainly regulated by other *PvSPL* subfamily genes. However, it is unclear if the PvSPLs functioning vegetative-to-floral phase transition can control internode numbers as well. Previous studies have shown that AtSPL3 can modulate floral phase transition in Arabidopsis [40]. Moreover, our finding revealed that the increased internode numbers were only observed in the transgenic switchgrass lines with greatly delayed flowering such as miR156 and *rPvSPL2* miR156 overexpressors. Therefore, the question of whether disruption of switchgrass orthologs of AtSPL3 can produce more internodes with normal length in switchgrass deserves to be elucidated in the future.

Lignin content is an important trait negatively affecting bioconversion of cell wall polysaccharides and forage digestibility [41–45]. Genetic modification of lignin biosynthesis in switchgrass can reduce cell wall recalcitrance and therefore increase ethanol production and forage digestibility [44, 45]. In this study, we found that suppression of PvSPL2 activity significantly reduced AcBr lignin content and altered lignin composition in semi-dwarf transgenic switchgrass lines. Due to the reduction in lignin accumulation, more fermentable sugar was released from the above transgenic switchgrass lines. In agreement with PvSPL2SRDX overexpressors, the miR156 highly overexpressing transgenic switchgrass lines also exhibit significantly reduced lignin content and increased fermentable sugar amount. The biomass of the above miR156 overexpressors, however, is dramatically reduced due to the severe repression on the expression of various miR156-targeted *PvSPLs* [29]. Therefore, our results open a new avenue for developing switchgrass germplasm with both low lignin and high biomass yield.

Numerous transcription factors from MYB, NAC, and WRKY families have been suggested to directly bind the motifs of promoter sequences of lignin genes and regulate lignin biosynthesis and secondary cell wall formation [46, 47]. In addition, the non-miR156-targeted AtSPL7 has been indicated to affect lignin accumulation through activation of certain microRNAs including miR397, miR408, and miR857 that can repress the expression of laccase genes in Arabidopsis [14, 19]. To date, there has been no report on participation of miR156-targeted *SPLs* in the regulation network of lignin biosynthesis. In contrast, miR156-targeted *SPLs* have been recently shown to regulate the biosynthesis of anthocyanin, sesquiterpene, and carotenoid. For example, Arabidopsis AtSPL9 negatively regulates anthocyanin accumulation through destabilization of an MYB–bHLH–WD40 transcriptional activation complex which plays a crucial role in regulating the expression of anthocyanin genes [16]. Moreover, AtSPL9 can bind to the *cis*-regulatory motifs of *terpene synthase 21* (*TPS21*) promoter both in vitro and in vivo and positively regulate sesquiterpene biosynthesis through activating *TPS21* expression [17]. Additionally, although the detailed regulation mechanism is not well understood yet, mutation of *AtSPL15* can clearly affect carotenoid biosynthesis in Arabidopsis [18]. In this study, we found the impaired expression of monolignol biosynthesis genes in PvSPL2SDRX overexpressors, suggesting that the miR156-targeted *PvSPLs*, at least the *PvSPL2* and its paralogs, can affect lignin biosynthesis in switchgrass. The hypothesis was further supported by the finding that the promoter sequences of switchgrass monolignol genes contained several SPL core binding sites. However, we cannot exclude the possibility that some specific interference between PvSPL2 and other transcriptional activation complexes may modulate the expression of monolignol genes. Therefore, the direct regulation mechanism of PvSPL2 on lignin biosynthesis deserves additional investigation in future.

## Conclusion

We found that the miR156-targeted *PvSPL1* and *2* modulated tiller initiation and stem elongation predominantly, whereas they had no effects on vegetative-to-floral phase transition and internode initiation. These results indicate that the different *PvSPL* subfamily genes are involved in different biological processes. Thus, functional characterization of each individual *PvSPL* will expedite the development of novel switchgrass germplasms with desirable traits by transgene pyramiding. Additionally, genetic manipulation of PvSPL2 in switchgrass can increase tiller numbers and reduce lignin accumulation, which consequently result in elevated biomass yield and cell wall saccharification efficiency. We suggest that PvSPL2 and its paralogs can be utilized as the valuable candidates in molecular breeding for design of new herbaceous bioenergy crop germplasms with high biomass yield and low cell wall recalcitrance.

## Methods

### Plant materials

A widely used and highly productive lowland-type switchgrass cultivar, Alamo, was used for genetic transformation and biomass improvement. Switchgrass plants were grown in the greenhouse with 16-h light (390 µE m^−2^ S^−1^). The development of switchgrass in our greenhouse was divided into three vegetative stages (V1, V2, and V3), five elongation stages (E1, E2, E3, E4, and E5), and three reproductive stages (R1, R2, and R3) according to the criteria described by Moore et al. [48].

### Sequence retrieval and identification

The conserved SBP domain based on Hidden Markov Model (HMM) (PF03110) was obtained from Pfam protein family database (http://pfam.sanger.ac.uk/), and then used as a query to search against the switchgrass genome database from Phytozome (http://www.phytozome.net/). Sequences were selected for further analysis if the *E* value was less than 1e^−10^ and confirmed by Pfam (PF03110). The coding regions of several *SPLs* were corrected according to the unique transcript sequence database of switchgrass [49]. All of the confirmed SPL proteins were aligned using Clustal X to manually remove the redundant sequences. In addition, peptide length, molecular weight, and isoelectric point of each PvSPL were calculated by online ExPasy program (http://www.expasy.org/).

### Phylogenetic analysis of *SPL* gene family

The putative PvSPL proteins and SPL proteins from another ten species were used to construct the phylogenetic tree. Sequences of SPL proteins of the six monocot species (switchgrass, foxtail millet, maize, sorghum, rice, and Brachypodium) were obtained from the public genome database Phytozome, while the sequences of Arabidopsis, citrus, cotton, grape, and poplar were extracted from GenBank according to the previous studies [20–24]. Multiple sequence alignments of the full-length SPL sequences were performed using Clustal W and manually trimmed the edges of the alignment. Unrooted maximum likelihood trees were constructed using LG model with aLRT SH-like branch support steps in PhyML version 3.0 which can improve tree likelihood and run faster than bootstrap [50] (http://atgc.lirmm.fr/phyml/) and then were manually improved by EvolView (http://www.evolgenius.info/evolview/).

### Chromosomal location and gene duplication of *PvSPLs*

Chromosome location was completed using MapChart 2.2 [51] based on the genetic linkage map constructed by Okada et al. [52]. Tandem gene duplication was defined as paralogous genes located within 50 kb in tandem and were separated by less than five non-homologous spacer genes [53].

### Gene structure analysis and conserved motif composition prediction

The exon/intron structures of PvSPLs were determined by comparing the CDS and corresponding genome sequences in the Gene Structure Display Server (GSDS, http://gsds.cbi.pku.edu.cn/). Conserved motifs were analyzed using the MEME program (http://meme.nbcr.net/meme/).

### Expression pattern analysis of *PvSPLs* and mature miR156 level in switchgrass

qRT-PCR was performed to analyze transcript abundance of *PvSPLs* in wild-type and miR156-overexpressing transgenic switchgrass plants. Total RNAs were extracted from the internodes and leaves along the tiller at E3 stage by Tri-Reagent (Invitrogen, Chicago, IL) and subjected to reverse transcription with Superscript III Kit (Invitrogen, Chicago, IL) after treatment with Turbo DNase I (Ambion, Austin, TX). SYBR Green (Applied Biosystems, Foster City, CA, USA) was used as the reporter dye. The primers used for qRT-PCR are listed in Additional file 7: Table S4. The cycle thresholds were determined using ABI PRISM 7900 HT sequence detection system (Applied Biosystems, Foster City, CA), and the data were normalized using the level of switchgrass *Ubq2* transcripts (HM209468). The mature miR156 level was detected and quantified by a highly sensitive quantitative RT-PCR method [54].

### Gene constructs and transformation

*PvSPL1* and *2* were isolated from switchgrass stem tissues by RT-PCR based on the sequence downloaded from Phytozome and subjected to sequencing. The primers used for the cloning of fragments of *PvSPL1/2SRDX* and* rPvSPL1/2* were designed based on the code sequences of the isolated *PvSPL1* and *2* (Additional file 7: Table S4). The final binary vectors of pANIC6B-PvSPL1/2SRDX and pANIC6D-rPvSPL1/2OE were constructed by LR recombination reactions (Invitrogen), and transferred into *Agrobacterium tumefaciens* strain *AGL1*.

A high-quality embryogenic callus line with single genotype established by screening numerous switchgrass Alamo seed-induced calli was employed for *Agrobacterium*-mediated transformation following the procedure described by Xi et al. [55]. Transgenic switchgrass lines and their corresponding empty vector control plants were grown in the greenhouse at 26 °C with 16-h light (390 µE m^−2^ S^−1^). In addition, the calli induced from nodes of the selected TmiR156OE9 transgenic line were used for the retransformation of pANIC6D-rPvSPL2OE construct into the miR156 overexpression background. Hygromycin and bialaphos were used as the selectable reagents to generate TrPvSPL2OEmiR156OE9 transgenic lines. The positive transgenic lines were identified by genomic PCR.

### Transcript abundance of *PvSPL1/2* in transgenic switchgrass plants

Total RNAs were isolated from the stem at E3 stage. The transcript abundances of Pv*SPL1/2* and *endo*-*PvSPL1/2* in PvSPL1/2SRDX-, rPvSPL1/2-, and rPvSPL2 miR156-overexpressing transgenic switchgrass lines were determined by qRT-PCR as described by Fu et al. [29].

### Development and growth analysis of transgenic switchgrass plants

The positive transgenic switchgrass plants were transported into the soil for morphological analysis. The controls were generated from a population including the transgenic plants with empty vector and false-positive plants. The flowering time of first five tillers was documented for transgenic and control plants. The number of total tillers was accounted for the plants after 4-month growth in the greenhouse. The 4-month-old tillers were used to measure plant height. The diameter of matured internodes (I3) of the above tillers was measured by calipers. The leaves adjacent to the node at the base of I3 were employed to measure leaf blade length and width. Transgenic and control plants were harvested after 4-month growth in greenhouse and dried in an oven at 40 °C for 96 h to evaluate the above-ground dry matter biomass yield.

### Determination of lignin content and composition

Stems of the transgenic switchgrass plants at the R1 stage were harvested. The collected samples were ground in liquid nitrogen and lyophilized. Lyophilized extractive-free cell wall residue (CWR) was used for lignin analysis. The AcBr method described by Hatfield et al. [56] was employed to quantify lignin content. The thioacidolysis method was used to determine lignin composition [57].

### Transcript abundance analysis of monolignol genes of PvSPL2SRDX transgenic switchgrass lines

Total RNAs from the internode 3 at the R1 stage of control and PvSPL2SRDX transgenic plants were isolated and subjected to reverse transcription. The transcript abundance of monolignol genes was analyzed by qRT-PCR. The primers used for qRT-PCR were designed as described by Shen et al. [33].

### Determination of sugar released by enzymatic hydrolysis

Cell wall residues generated for lignin analysis were also used to analyze sugar release efficiency and total solubilized sugar amount according to previously described procedures [29]. Sugar release was analyzed spectrophotometrically using the phenol–sulfuric acid assay method [58]. Saccharification efficiency was determined as the ratio of sugars released by enzymatic hydrolysis to the amount of sugars present in the cell wall material before pretreatment. Total solubilized sugar produced by acid pretreatment followed enzymatic hydrolysis was calculated by combining the biomass and sugar release efficiency [29].

### Statistical analysis

Triplicate samples were collected for each control and transgenic plant. Data from each trait were subjected to analysis of variance (ANOVA). The significance of treatments was tested at the *P* < 0.05 level. Standard errors were provided in all tables and figures as appropriate. All the statistical analyses were performed with the SPSS package (SPSS Inc., Chicago, IL, USA).

## Additional files

10.1186/s13068-016-0516-z Molecular characterization of PvSPLs.
10.1186/s13068-016-0516-z List of SPL sequences for phylogenetic analysis.
10.1186/s13068-016-0516-z Molecular characterization of *PvSPL* genes. **a** Exon-intron structures of *PvSPLs*. Exon and intron are represented by box and line, respectively. The open reading frames (ORFs) are shown in yellow, and the untranslated region (UTR) are shown in blue. The scale at the bottom indicates the sizes of *PvSPL* genes. **b** Schematic representation of conserved motifs in PvSPL proteins predicted by MEME. Each motif is represented by a colored box, and the black lines represent non-conserved sequences. **c** Motif logos of PvSPL conserved domains.
10.1186/s13068-016-0516-z Relationships between expression levels of the miR156-targeted *PvSPL* genes and tiller numbers. Twelve independent positive miR156 overexpressing transgenic switchgrass lines were generated by *Agrobacterium-*mediated transformation. Expression levels of *PvSPL1/19*, *2/18*, *3/5*, and *6/7* in the transgenic switchgrass plants were detected by qRT-PCR. Switchgrass *Ubq2* was used as the reference for normalization.
10.1186/s13068-016-0516-zExpression levels of *PvSPL1* in PvSPL1SRDX and rPvSPL1 overexpressing transgenic switchgrass plants were revealed by qRT-PCR. Switchgrass *Ubq2* was used as the reference for normalization. *PvSPL1*: sum of *exo*- and *endo*-*PvSPL1* transcript versions.
10.1186/s13068-016-0516-z Morphological characterization of PvSPL1SRDX overexpressing transgenic switchgrass plants.
10.1186/s13068-016-0516-z Primer sequences used for gene expression analysis by qRT-PCR and plasmid construction.

## Abbreviations

AcBr: acetyl bromide
ANOVA: analysis of variance
*CAD*: *cinnamyl alcohol dehydrogenase*
*CCR1*: *cinnamoyl CoA reductase 1*
Chr: chromosome
*4CL1*: *4-coumarate:CoA ligase 1*
*COMT*: *caffeic acid O-methyltransferase*
CRES-T: chimeric repressor gene silencing technology
CWR: cell wall residue
*F5H*: *ferulate 5-hydroxylase*
G: guaiacyl
miR156: microRNA156
ML: maximum likelihood
OG: orthologous group
qRT-PCR: quantitative reverse transcription polymerase chain reaction
S: syringyl
SBP: SQUAMOSA PROMOTER-BINDING PROTEIN
SPL: SQUAMOSA PROMOTER-BINDING PROTEIN-LIKE
