# Supplementary figures and images for: Switchgrass SBP-box transcription factors PvSPL1 and 2 function redundantly to initiate side tillers and affect biomass yield of energy crop
Source: Biotechnol Biofuels. 2016 May 5;9:101. doi: 10.1186/s13068-016-0516-z (PMC4858904; doi:10.1186/s13068-016-0516-z)

## Slide 1
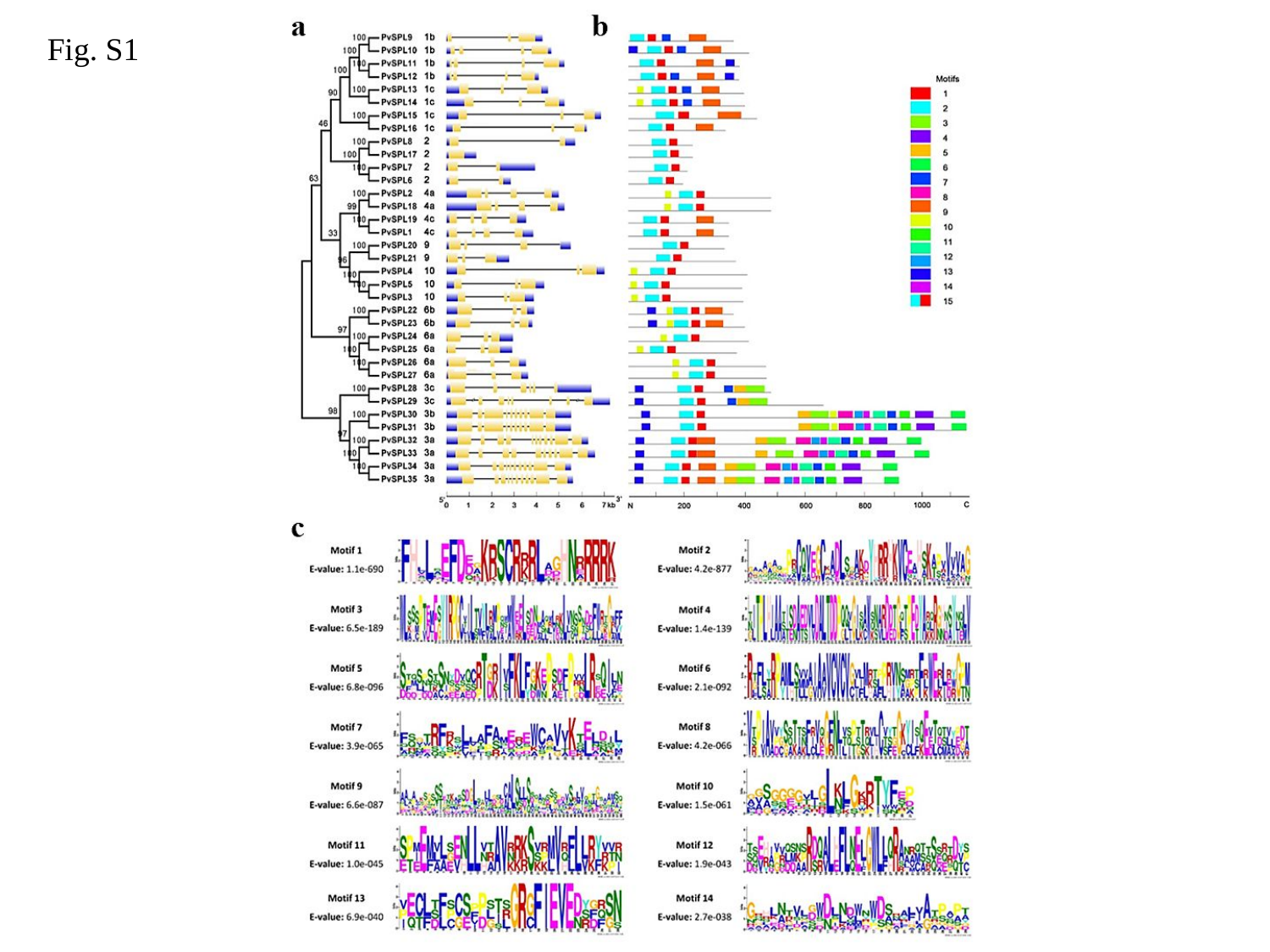

Fig. S1

Supplement: Supplementary file 3 — 10.1186/s13068-016-0516-z Molecular characterization of PvSPL genes. a Exon-intron structures of PvSPLs. Exon and intron are represented by box and line, respectively. The open reading frames (ORFs) are shown in yellow, and the untranslated region (UTR) are shown in blue. The scale at the bottom indicates the sizes of PvSPL genes. b Schematic representation of conserved motifs in PvSPL proteins predicted by MEME. Each motif is represented by a colored box, and the black lines represent non-conserved sequences. c Motif logos of PvSPL conserved domains. [file 13068_2016_516_MOESM3_ESM.ppt]

## Slide 1
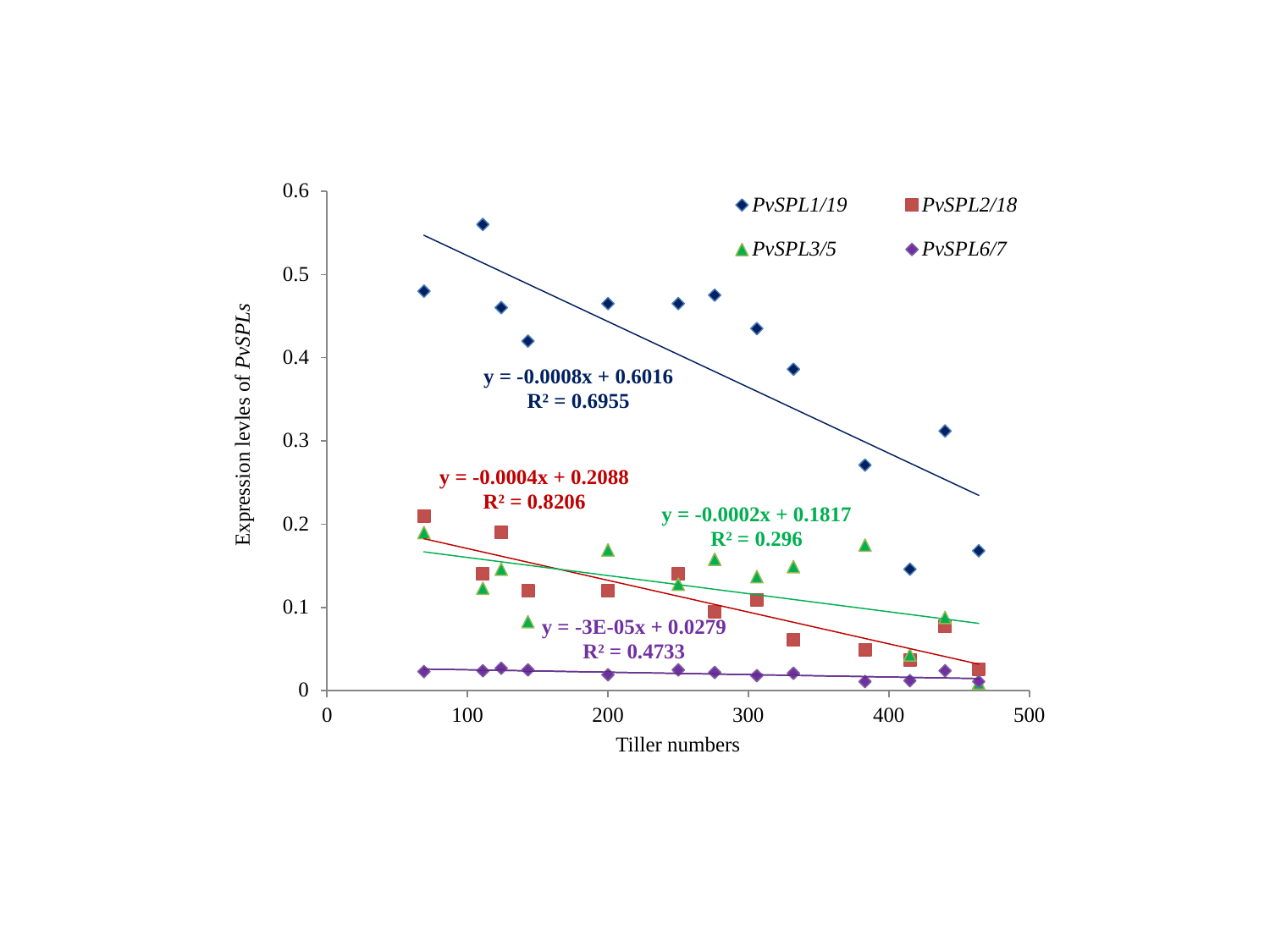

Supplement: Supplementary file 4 — 10.1186/s13068-016-0516-z Relationships between expression levels of the miR156-targeted PvSPL genes and tiller numbers. Twelve independent positive miR156 overexpressing transgenic switchgrass lines were generated by Agrobacterium-mediated transformation. Expression levels of PvSPL1/19, 2/18, 3/5, and 6/7 in the transgenic switchgrass plants were detected by qRT-PCR. Switchgrass Ubq2 was used as the reference for normalization. [file 13068_2016_516_MOESM4_ESM.ppt]

## Slide 1
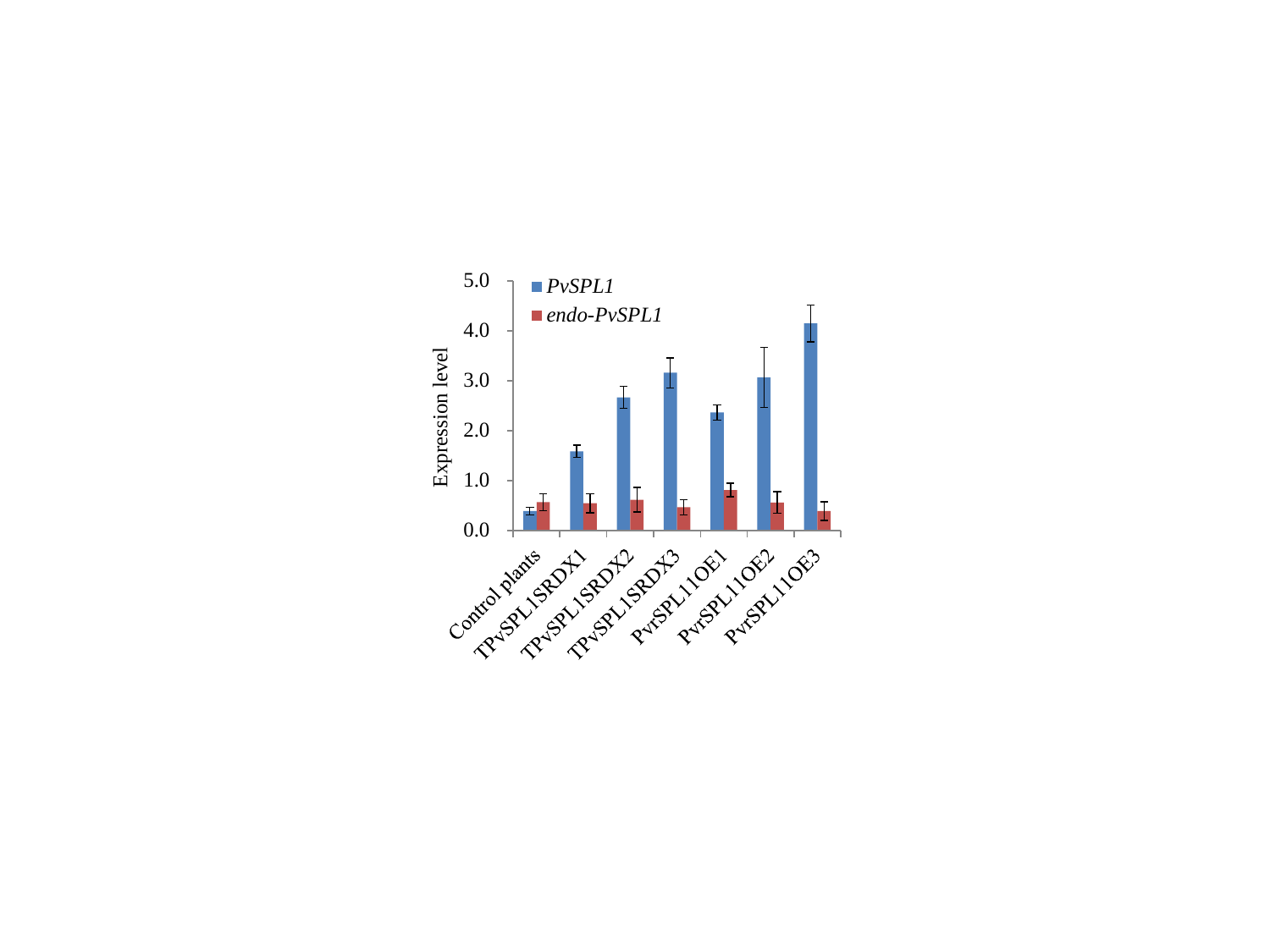

Supplement: Supplementary file 5 — 10.1186/s13068-016-0516-zExpression levels of PvSPL1 in PvSPL1SRDX and rPvSPL1 overexpressing transgenic switchgrass plants were revealed by qRT-PCR. Switchgrass Ubq2 was used as the reference for normalization. PvSPL1: sum of exo- and endo-PvSPL1 transcript versions. [file 13068_2016_516_MOESM5_ESM.ppt]
