# Supplementary material for: Switchgrass SBP-box transcription factors PvSPL1 and 2 function redundantly to initiate side tillers and affect biomass yield of energy crop
Source: Biotechnol Biofuels. 2016 May 5;9:101. doi: 10.1186/s13068-016-0516-z (PMC4858904; doi:10.1186/s13068-016-0516-z)
Supplement: Supplementary file 6 — 10.1186/s13068-016-0516-z Morphological characterization of PvSPL1SRDX overexpressing transgenic switchgrass plants. [file 13068_2016_516_MOESM6_ESM.doc]

Table S3 Morphological characterization of PvSPL1SRDX and rPvSPL1 overexpressing transgenic switchgrass plants.

|  | Plant height (cm) | Tiller number | Internode length (cm) | Internode number | Flowering time (day) |
| --- | --- | --- | --- | --- | --- |
| Control plants | 104.2±9.7 | 16.7±0.9 | 16.4±0.5 | 4-5 | 101±3 |
| TPvSPL1SRDX1 | 110.3±5.0 | 27.0±1.2* | 17.3±0.9 | 4-5 | 98±6 |
| TPvSPL1SRDX2 | 83.2±2.1* | 35.7±1.2* | 13.1±1.3* | 4-5 | 96±3 |
| TPvSPL1SRDX3 | 80.6±4.3* | 37.9±0.6* | 12.9±1.1* | 4-5 | 99±4 |
| TrPvSPL1OE1 | 117.4±10.4 | 6.3±1.2* | 15.6±0.3 | 4-5 | 103±2 |
| TrPvSPL1OE2 | 106.5±3.7 | 8.7±0.9* | 16.4±1.3 | 4-5 | 98±2 |
| TrPvSPL1OE3 | 98.7±2.4 | 6.3±0.3* | 17.0±0.7 | 4-5 | 100±3 |

^†^Plant height and tiller numbers of switchgrass were measured after 4-month growth in the greenhouse. The 4-month-old tillers were used to measure internode length (internode 3), internode number and flowering time. Five tillers were measured for each replicate. Control plants were produced with pANIC6B and 6D empty vectors, respectively. Values are mean ± SE (n=3). Asterisk indicates significance corresponding to P < 0.05 (One way ANOVA, Dunnett’s test).
